# The G protein-coupled receptor GPR157 regulates neuronal differentiation of radial glial progenitors through the Gq-IP_3_ pathway

**DOI:** 10.1038/srep25180

**Published:** 2016-05-04

**Authors:** Yutaka Takeo, Nobuhiro Kurabayashi, Minh Dang Nguyen, Kamon Sanada

**Affiliations:** 1 Department of Biophysics and Biochemistry, Graduate School of Science, The University of Tokyo, Hongo 7-3-1, Bunkyo-ku, Tokyo 113-0033, Japan; 2 Molecular Genetics Research Laboratory, Graduate School of Science, The University of Tokyo, Hongo 7-3-1, Bunkyo-ku, Tokyo 113-0033, Japan; 3 Hotchkiss Brain Institute, University of Calgary, Departments of Clinical Neurosciences, Cell Biology & Anatomy, Biochemistry & Molecular Biology, Calgary, Alberta, T2N4N1 Canada

## Abstract

The ability of radial glial progenitors (RGPs) to generate cortical neurons is determined by local extracellular factors and signaling pathways intrinsic to RGPs. Here we find that GPR157, an orphan G protein-coupled receptor, localizes to RGPs’ primary cilia exposed to the cerebrospinal fluid (CSF). GPR157 couples with Gq-class of the heterotrimeric G-proteins and signals through IP_3_-mediated Ca^2+^ cascade. Activation of GPR157-Gq signaling enhances neuronal differentiation of RGPs whereas interfering with GPR157-Gq-IP_3_ cascade in RGPs suppresses neurogenesis. We also detect the presence of putative ligand(s) for GPR157 in the CSF, and demonstrate the increased ability of the CSF to activate GPR157 at neurogenic phase. Thus, GPR157-Gq signaling at the primary cilia of RGPs is activated by the CSF and contributes to neurogenesis.

Nearly all adult excitatory cortical neurons derive from radial glial progenitors (RGPs) located in the ventricular zone (VZ) of the developing neocortex^1^,^2^,^3^. During early neocortical development, RGPs divide symmetrically to amplify the progenitor pools. As corticogenesis proceeds, RGPs divide asymmetrically to self-renew and to produce neurons either directly, or indirectly through intermediate progenitors^4^,^5^. Fate determination of RGPs results from integration of signals borne by a variety of sources (i.e. neighboring neurons, neighboring RGPs, meninges, and cerebrospinal fluid−CSF) to cell-intrinsic programs^6^,^7^,^8^.

RGPs facing the lateral ventricle expose their primary cilia to the CSF. As cerebral hypoplasia is caused by ciliary dysfunction^9^, RGPs’ primary cilia are important for normal neocortical development^10^,^11^. As sensors of the environment, they can transduce signals originating from the CSF (such as Sonic Hedgehog) into RGPs^10^. Interestingly, the composition of the CSF changes throughout neurodevelopment^12^ and consequently could impact RGPs biology in a neurogenic/gliogenic phase-dependent manner.

G protein-coupled receptors (GPCRs) constitute the largest family of membrane receptors that transduce environmental signals into the cell through activation of cognate heterotrimeric G-proteins. Generally, heterotrimeric G-proteins are divided into four families based on their sequence similarity, Gs, Gi, Gq, and G12/13 families^13^. Noticeably, GPCR-mediated signaling has recently been implicated in several aspects of RGPs biology. For instance, GPR56 contributes to the proper interaction of RGPs with the basement membrane while its mutations are associated with a human brain malformation called bilateral frontoparietal polymicrogyria^14^,^15^. Depletion of GPRC5B, an orphan GPCR expressed in RGPs, gives rise to cells that eventually differentiate into astrocytes^16^. Moreover, perturbation of Gαi proteins affects proliferation of RGPs in the developing neocortex^17^. Finally, activation of the Gq-IP_3_ pathway via certain GPCRs leads to proliferation of RGPs^18^,^19^.

Despite these findings, it remains unknown whether GPCR signaling can direct neuronal differentiation of RGPs in the developing neocortex. Furthermore, the potential involvement of GPCR signaling at the RGPs-CSF interface has not been examined. In this study, we discovered that the orphan GPCR GPR157 is expressed in the primary cilia of RGPs, responds to constituent(s) of the CSF, and promotes neuronal differentiation of RGPs via the Gq-IP_3_ pathway.

## Results

### GPR157 is located in the primary cilia of RGPs in the developing neocortex

**Figure 1:**
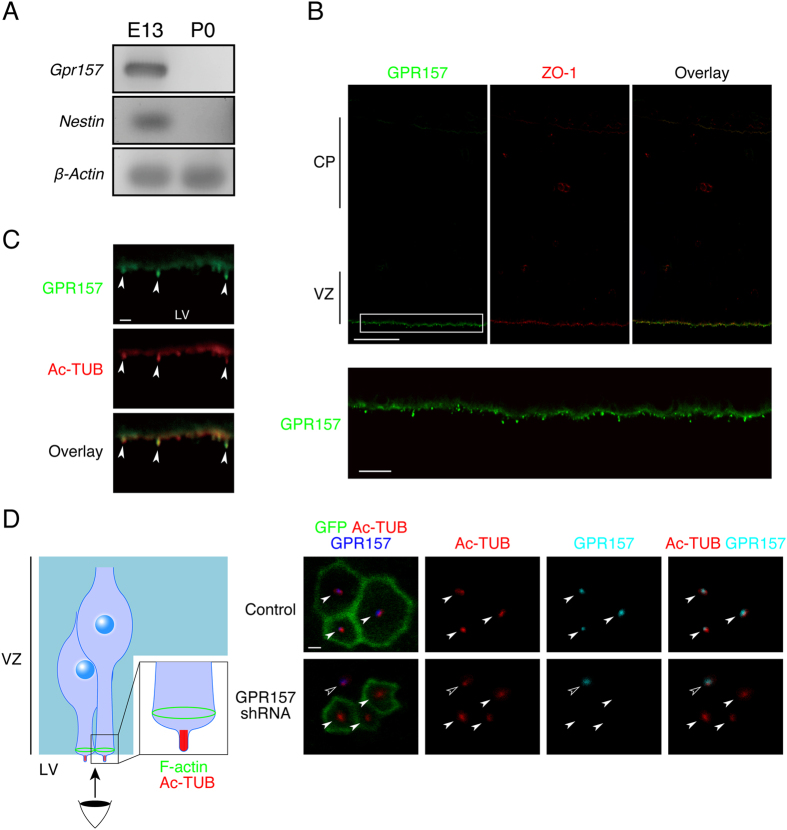
GPR157 is expressed in primary cilia of RGPs in the developing neocortex. (**A**) Expression of *Gpr157* was analysed by RT-PCR using cDNAs derived from mouse neocorticies at E13 and P0. *Nestin* is a marker of RGPs. (**B**) E13 neocortical coronal sections immunostained with anti-GPR157 (green) and anti-ZO-1 (red) antibodies. Images of the entire cerebral wall are shown (upper panels). Magnified image of the boxed area in the upper panel was shown below. VZ, ventricular zone. CP, cortical plate. (**C**) High magnification images around the ventricular surface of E13 neocortical sections immunostained with anti-GPR157 (green) and anti-Ac-TUB (red) antibodies. Arrowheads indicate Ac-TUB-positive primary cilia protruding from the ventricular surface. (**D**) Left, Schematic diagram of en face view imaging. Right, High-magnification images of en face view of the ventricular surface of neocortices electroporated with either Lifeact-EGFP (upper panels) or Lifeact-EGFP/GPR157 shRNA (lower panels). The cortices were stained with antibodies against GPR157 and Ac-TUB. Closed and open arrowheads indicate primary cilia in transfected and non-transfected cells, respectively. LV: lateral ventricle, VZ: ventricular zone. Scale bars: 50 μm in (**B**) (upper panel); 10 μm in (**B**) (lower panel); 1 μm in (**C**,**D**).

GPR157 is an orphan GPCR whose functions have not been explored to date. PCR analysis with cDNAs derived from the embryonic day 13 (E13) and postnatal day 0 (P0) mouse neocortex revealed that the mRNA of *Gpr157* was hardly detectable in the P0 neocortex whereas its expression was robust at E13 (Fig. 1A). To localize GPR157 protein in the developing neocortex, an affinity-purified rabbit anti-GPR157 antibody was generated. The antibody specifically detected GPR157 transiently expressed in U-2 OS cells, as assessed by western blotting (Supplementary Fig. S1). When brain coronal sections were immunostained with this antibody, GPR157 immunofluorescent signals were detected around the luminal surface of the ventricular zone (VZ) in the developing neocortex at E13 (Fig. 1B). Detailed immunohistochemical analyses revealed that punctate GPR157 immunofluorescent signals were detected outside of the ventricular surface. Noticeably, they overlapped with staining signals of acetylated tubulin (Ac-TUB; Fig. 1C) that is assembled in the axonemes of the primary cilia of RGPs in the developing neocortex^20^. Almost no GPR157 immunofluorescent signals were detected in sections immunostained with antibody pre-incubated with its immunogen (Supplementary Fig. S2). In addition, GPR157 signals were not detected in regions enriched with TBR2-positive intermediate progenitors^21^ and βIII-tubulin (TUJ1)-positive neurons (Supplementary Fig. S3). These results suggest that GPR157 is enriched in RGPs and unlikely expressed in intermediate progenitors/neurons.

We went on to study the developmental expression of GPR157 in E10, E17, P0 and adult neocortex. At E10, GPR157 immunofluorescent signals showed significant overlap with Ac-TUB signals at the ventricular surface, similar to those at E13 (Supplementary Fig. S4). In E17, P0, and adult neocortex, no GPR157 signal was observed at the ventricular surface but also in the entire cerebral wall (Supplementary Fig. S4). This result is consistent with the undetectable expression of *Gpr157 *mRNA in the P0 neocortex and suggests preferential expression of GPR157 during the early to mid stages of corticogenesis.

Given that GPR157 immunoreactivity overlapped with Ac-TUB, the en face view of the ventricular surface of neocortices stained with GPR157 antibody was imaged in order to refine the expression pattern of GPR157 in the primary cilia of RGPs. For this, E13 neocortices were electroporated with Lifeact-EGFP (MGVADLIKKFESISKEE-EGFP) to label F-actin that is assembled in the adherens junctions within the apical side of the descending processes of RGPs^22^ (see also the diagram in Fig. 1D). The cortices were then immunostained with antibodies against GPR157 and Ac-TUB. The en face view showed a honeycomb-like organization of F-actin. Ac-TUB immunofluorescent signals were detected around the center of F-actin rings. GPR157 immunoreactivity shows significant overlap with Ac-TUB signals. Of note, GPR157 immunoreactivity appears to not uniformly localize along the entire length of the primary cilia. This raises the possibility that GPR157 is enriched in specific subdomains in the cilia. Such enrichment has been previously observed for several certain ciliary membrane^23^. Specificity of the GPR157 antibody for staining of brain sections was confirmed with brains electroporated with the small hairpin RNA (shRNA) against *Gpr157* transcript (see below). While Ac-TUB and F-actin were clearly detected in GPR157 shRNA-introduced cells, GPR157 signals were remarkably diminished (Fig. 1D). Together, these results indicate that GPR157 localizes to the primary cilia of RGPs in the developing neocortex.

### GPR157 knockdown inhibits neuronal differentiation of RGPs

**Figure 2:**
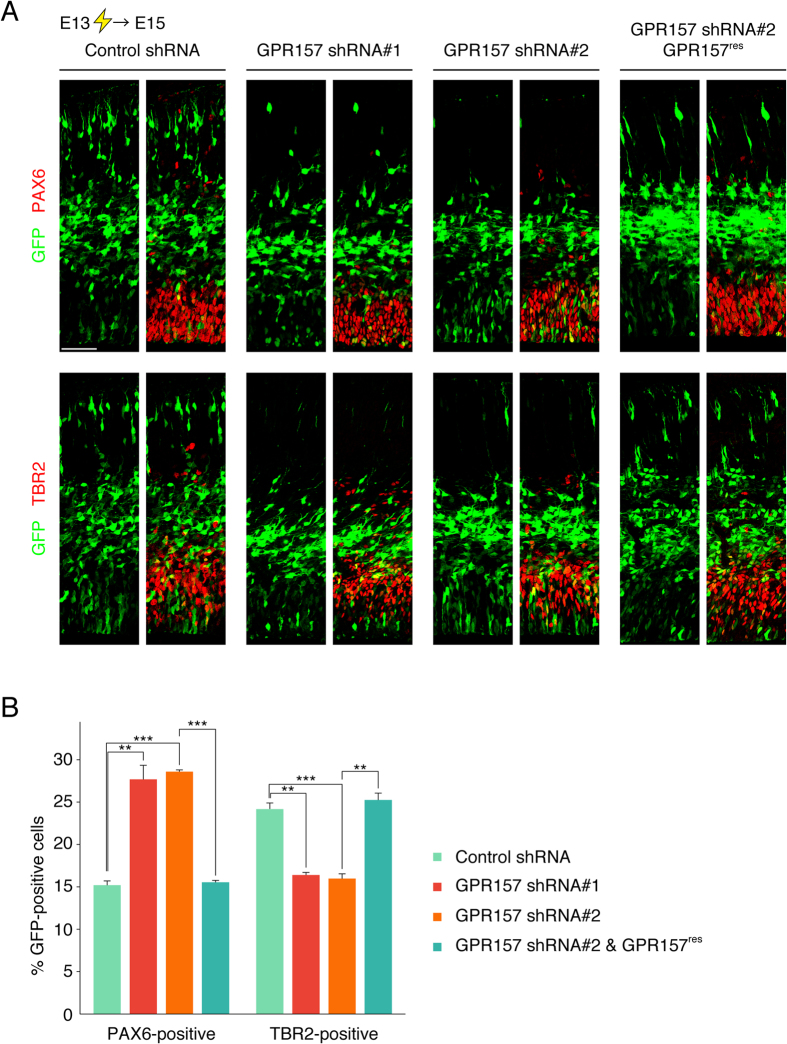
GPR157 knockdown impairs neuronal differentiation of RGPs. (**A**) Plasmids expressing control shRNA, GPR157 shRNA#1, GPR157 shRNA#2 or both GPR157 shRNA#2 and GPR157^res^ (GPR157 that contains two silent mutations within the GPR157 shRNA#2-targeted sequence) were electroporated, together with the GFP-expressing plasmid, into E13 embryos. E15 brain sections were immunostained with antibodies against PAX6 (upper panels) or TBR2 (lower panels). Images of the entire cerebral wall are shown. (**B**) The graphs show the fraction of GFP-positive cells that were also positive for PAX6 or TBR2. Data are presented as mean ± s.e.m. (n = 3). **p < 0.01, ***p < 0.001. Scale bar: 50 μm.

We investigated a potential role for GPR157 in RGPs of developing neocortex (Fig. 2). For this, we generated shRNA expression constructs targeting *Gpr157* transcript. We identified two different shRNA constructs (shRNA#1, shRNA#2) capable of knocking down expression of GPR157 transiently expressed in U-2 OS cells (Supplementary Fig. S1). Contrariwise, expression of GPR157 with two silent mutations within the target sequence of shRNA#2 (GPR157^res^) was not affected by the shRNA (Supplementary Fig. S1).

We then co-introduced either control or GPR157 shRNA constructs with the GFP-expression plasmid, into the VZ of the E13 neocortex by in utero electroporation, and harvested the embryos 48 hours later (at E15). In GPR157 shRNA-electroporated neocortices, a larger population of GFP-positive cells remained PAX6-positive RGPs when compared with the population found in control shRNA-expressing neocortices (Fig. 2). When RGPs were pulse-labeled with 5-bromo-2-deoxyuridine (BrdU) for 30 minutes, the BrdU-positive fraction of GFP-labeled PAX6-positive RGPs was not significantly changed upon introduction of GPR157 shRNAs (Supplementary Fig. S5). These results suggest that the increased population of RGPs upon introduction of GPR157 shRNA is not due to enhanced proliferation but rather to impaired differentiation.

To determine whether neuronal differentiation of progenitors is impaired upon GPR157 knockdown, we assessed the production of intermediate progenitors by immunohistochemistry using an antibody against TBR2. We found a significant reduction in the fraction of TBR2-positive intermediate progenitors in GPR157 shRNA-introduced neocortices when compared to control shRNA-expressing neocortices (Fig. 2). Altogether, these observations suggest that introduction of the GPR157 shRNA in RGPs impairs their neuronal differentiation in the developing neocortex. To confirm that impaired neurogenesis results specifically from GPR157 knockdown, we performed a rescue experiment by overexpressing GPR157^res^. Expression of GPR157 mutant almost completely reversed the phenotypes caused by GPR157 shRNA#2, i.e. the abnormal increase in PAX6 population and decrease in TBR2 fraction (Fig. 2). There was no significant difference in the percentage of apoptotic cells among the GFP-positive cells at any condition (<1%), thereby excluding apoptosis as an explanation for the observed phenotypes. Taken together, these results confirm that impaired neurogenesis in GPR157 shRNA-introduced RGPs is caused by loss of GPR157 function.

We also examined the fate of GPR157-depleted cells at later neurodevelopmental stages. For this, we co-electroporated GFP-expressing plasmid with either control or GPR157 shRNA constructs at E14, and embryos were harvested at E17. In control neocortices, the majority of GFP-labeled cells were present in the cortical plate (CP) and the intermediate zone (IZ). In GPR157 shRNA-electroporated neocortices, a significantly larger population of GFP-positive cells was observed in the IZ and VZ while a fewer fraction was in the CP when compared to populations found in control neocortices (Supplementary Fig. S6). We next determined whether the redistribution of cells is correlated with altered neuronal differentiation using immunostaining with TUJ1 antibody. We found a significant reduction in the fraction of TUJ1-positive neurons in GPR157 shRNA-introduced neocortices (Supplementary Fig. S6). Altogether, these observations suggest that GPR157 depletion in RGPs results in a decreased propensity of RGPs to differentiate into intermediate progenitors (Fig. 2) and consequently neurons (Supplementary Fig. S6). As almost all GFP-labeled cells in the VZ of GPR157 shRNA-introduced neocortices were negative for TUJ1 and a sizable fraction of the GFP-labeled cells were in the CP, redistribution of these cells is unlikely due to migration defects of neurons.

### GPR157 couples with Gq-class of heterotrimeric G-proteins

**Figure 3:**
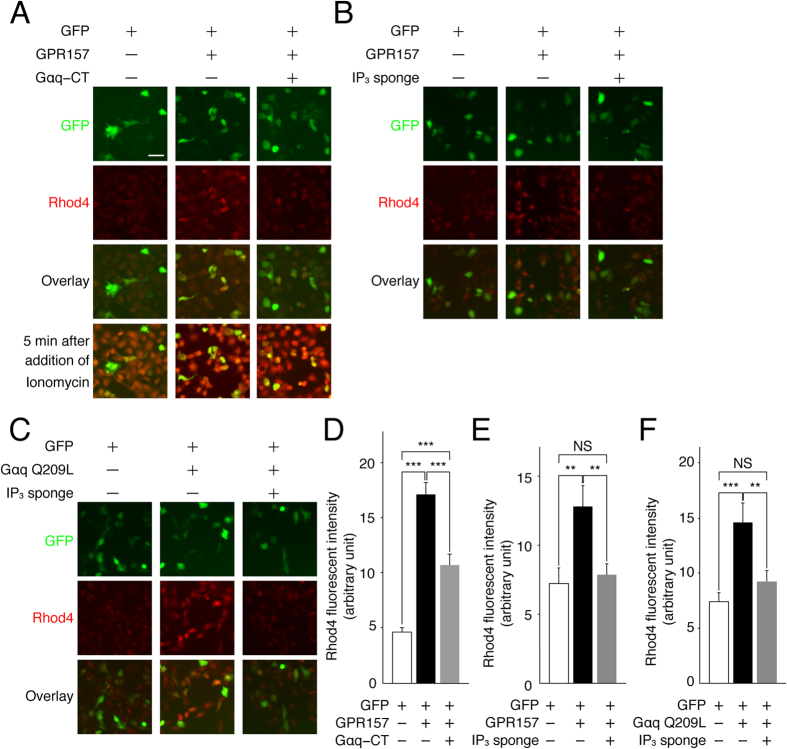
GPR157 couples with Gq-class of the heterotrimeric G-proteins. (**A**–**C**) Plasmids expressing indicated protein were transfected into U-2 OS cells. Rhod4, a fluorescent calcium indicator, were used to assess changes in [Ca^2+^]_i_. Application of Ionomycin, a calcium ionopohore, to cells after experiments confirmed almost uniform uptake of Rhod4 in these cells. (**D–F**) Fluorescent intensity of Rhod4 in GFP-positive cells. Mean ± s.e.m. Data were obtained from 3 independent experiments (more than 40 cells). **p < 0.01, ***p < 0.001. Scale bar: 10 μm.

Most of GPCRs transduce extracellular signals into cells via activation of heterotrimeric G-proteins. Gα protein family can be grouped into four subfamilies (Gαs, Gαi, Gαq, Gα12/13) with distinct signaling pathways^13^. It is known that overexpression of GPCRs leads to ligand-independent activation of their cognate G-proteins^24^. We found that overexpression of GPR157 induces an increase in intracellular calcium concentration ([Ca^2+^]_i_) in U-2 OS cells (Fig. 3A,D). We wanted to determine whether of ectopic GPR157 has similar effects on [Ca^2+^]_i_ in cultured RGPs. Following in utero co-electroporation of E13 RGPs with either control or GPR157-expressing plasmid and GFP-expressing plasmid, GFP-labeled cells were cultured and changes in [Ca^2+^]_i_ in these cells were measured 24 hours later. We found that overexpression of GPR157 induces an increase in [Ca^2+^]_i_ in GFP-labeled cells (Supplementary Fig. S7). Since almost all GFP-labeled cells (>96%) were SOX2-positive RGPs (Supplementary Fig. S7), we concluded that overexpression of GPR157 increase [Ca^2+^]_i_ in RGPs, like in U-2 OS cells.

Since the major downstream pathway of Gαq converges to Ca^2+^ release from intracellular Ca^2+^ stores through the activation of the PLC-IP_3_ pathway^25^, we determined whether GPR157-evoked increase in [Ca^2+^]_i_ is mediated via Gq-signaling. Peptides corresponding to C-terminal regions of Gα (Gα-CTs) can act as competitive blockers for GPCR-G protein signaling as they selectively bind to the G protein-binding site on cognate GPCRs and attenuate the signaling through a given G protein^26^. When Gαq-CT was co-expressed with GPR157, GPR157-induced augmentation in [Ca^2+^]_i_ was partially prevented (Fig. 3A,D). These observations indicate that GPR157 is coupled with Gq-class of heterotrimeric G-proteins.

To determine whether [Ca^2+^]_i_ elevation elicited by GPR157-Gq was mediated by IP_3_, we used IP_3_ trapping protein (IP_3_ sponge) to sequester IP_3_ and suppress IP_3_ receptor-mediated Ca^2+^ release^27^. When the IP_3_ sponge was co-expressed with GPR157, GPR157-induced elevation in [Ca^2+^]_i_ was almost completely suppressed (Fig. 3B,E). Like overexpression of GPR157, introduction of constitutively active Gαq (Gαq Q209L) in U-2 OS cells increased [Ca^2+^]_i_. Such increase was almost completely suppressed with co-expression of IP_3_ sponge (Fig. 3C,F). Altogether, these results suggest that GPR157 couples with Gq and activates PLC-IP_3_ pathway, thereby causing increase in [Ca^2+^]_i_.

### Gq and IP_3_ regulates neuronal differentiation of RGPs

**Figure 4:**
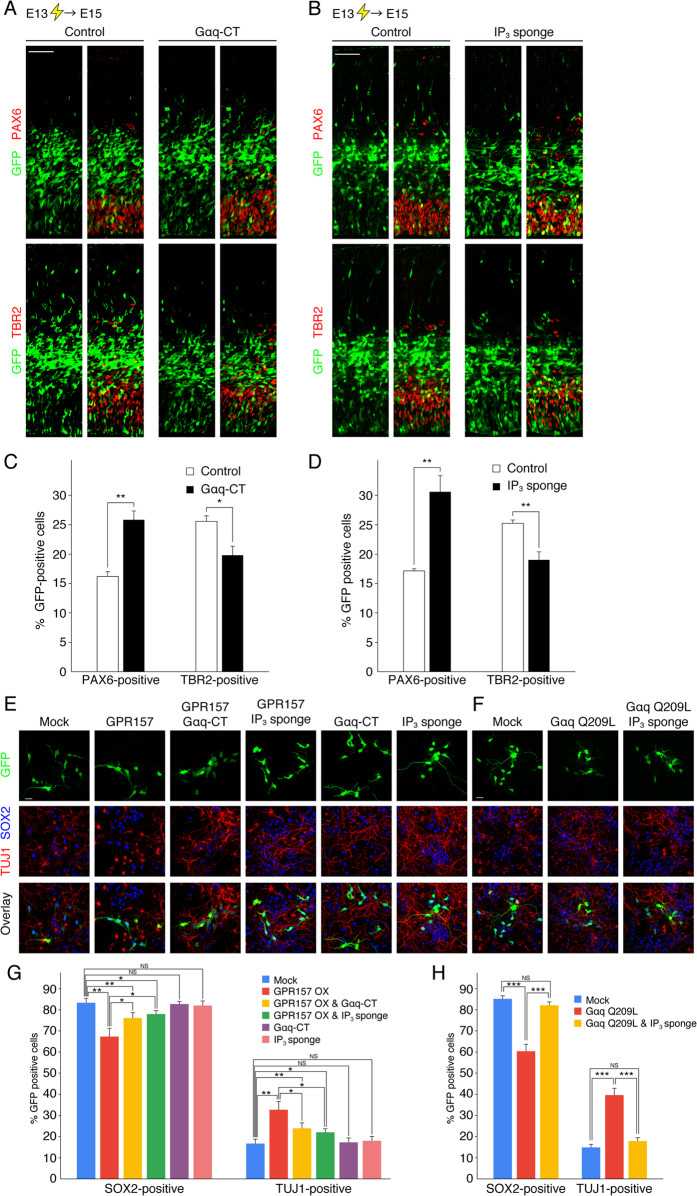
GPR157-Gq-IP_3_ signaling regulates neurogenesis of RGPs. (**A**) Plasmid expressing either Gαq-CT (right) or control plasmid (left) was electroporated, together with the GFP-expressing plasmid, into E13 embryos. E15 brain sections were immunostained with antibodies against PAX6 or TBR2. (**B**) Plasmid expressing either IP_3_ sponge (right) or control plasmid (left) was electroporated, together with the GFP-expressing plasmid, into E13 embryos. E15 brain sections were immunostained with antibodies against PAX6 or TBR2. (**C,D**) The graphs show the fraction of GFP-positive cells that were also positive for PAX6 or TBR2 in (**A**,**B**) respectively. Data are presented as mean ± s.e.m. (n = 3). *p < 0.05, **p < 0.01. Scale bars: 50 μm. (**E**) Plasmids expressing GPR157, both GPR157 and Gαq-CT, both GPR157 and IP_3_ sponge, Gαq-CT, or IP_3_ sponge were electroporated, together with the GFP-expressing plasmid, into E13 embryos. Cortical cells were prepared, cultured for 3 days, and immunostained with antibodies against SOX2 and TUJ1. (**F**) Plasmids expressing Gαq Q209L or both Gαq Q209L and IP_3_ sponge were electroporated, together with the GFP-expressing plasmid, into E13 embryos. Cortical cells at 3DIV were immunostained as in (**E**). (**G,H**) The graph showing the fraction of GFP-positive cells that were also positive for SOX2 or TUJ1 in (**E**) and (**F**) respectively. Data are presented as mean ± s.e.m. (n = 4; more than 100 cells were analyzed in each condition). *p < 0.05, **p < 0.01, ***p < 0.001. Scale bars: 50 μm in (**A**,**B)**; 20 μm in (**E,F**).

Given that GPR157 signals through Gq-IP_3_-mediated signaling (Fig. 3), we next examined whether alterations in Gq-IP_3_-mediated signaling recapitulate the effects of GPR157 depletion *in vivo*. Similarly to GPR157 knockdown, introduction of Gαq-CT or IP_3_ sponge increased the fraction of PAX6-positive cells and decreased the fraction of TBR2-positive cells when compared to controls (Fig. 4A–D). In addition, the BrdU-positive fraction of GFP-labeled PAX6-positive RGPs was not significantly changed by Gαq-CT and IP_3_ sponge, indicating that cell cycle progression of RGPs was not affected (Supplementary Fig. S8).

Next, we asked whether GPR157 signaling regulates neuronal differentiation of cultured RGPs. For this purpose, E13 embryos were co-electroporated with GFP and GPR157, cultured for 3 days, and immunostained with antibodies against TUJ1 and SOX2. The vast majority (85%) of control GFP-labeled cells were SOX2-positive RGPs, and the remaining cells (15%) were TUJ1-positive neurons. On the other hand, GPR157 overexpression gave rise to more than 30% of TUJ1-positive neurons (Fig. 4E,G). Given that neuronal differentiation (Fig. 4E,G) and [Ca^2+^]_i_ in cultured RGPs (Supplementary Fig. S7) were enhanced upon activation of GPR157 signaling, we asked the question whether GPR157, Gq, and IP_3_ regulate neuronal differentiation through the same pathway. To do so, we looked at the effects of Gαq-CT and IP_3_ sponge on GPR157-mediated neuronal differentiation. As shown in Fig. 4E,G, the increased production of neurons upon GPR157 overexpression was partially but significantly reversed by co-expression of Gαq-CT and IP_3_ sponge. Moreover, nearly 40% of Gαq Q209L-expressed cells were TUJ1-positive whereas the effect was counterbalanced by co-expression of IP_3_ sponge (Fig. 4F,H). When the cultures were pulse labeled with BrdU, no significant difference in populations of BrdU-positive cells among GFP- and SOX2-postive cells was found at any condition (Supplementary Fig. S9). Also, there was no significant difference in the percentage of apoptotic cells among the GFP-positive cells at any condition (<1%). These results indicate that GPR157-Gq-IP_3_ signaling contributes to neuronal differentiation of RGPs.

### Putative ligand(s) of GPR157 are present in the CSF

**Figure 5:**
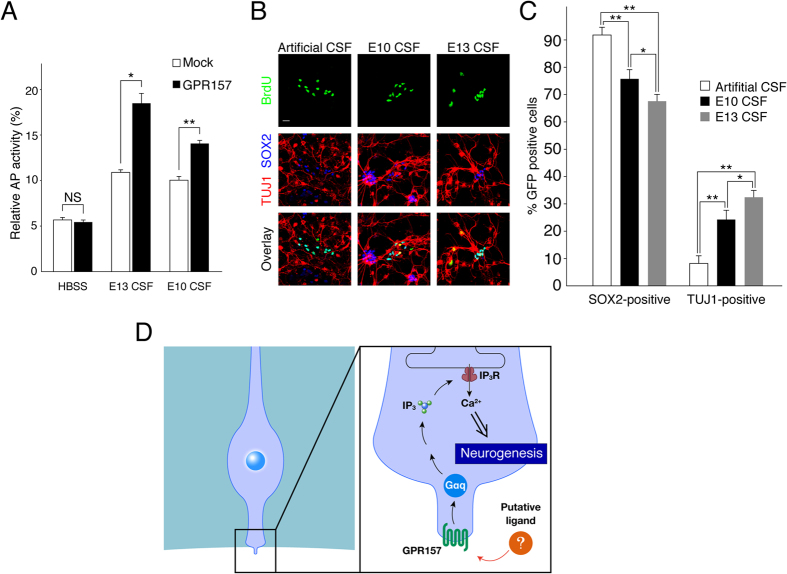
Putative ligand of GPR157 is present in the CSF at E13. (**A**) AP-TGFα-expressing plasmid was transfected into U-2 OS cells, with (GPR157) or without the GPR157-expressing plasmid. Relative AP activity was measured after treatment of the cells with either HBSS, E13 CSF (1/8 dilution with HBSS), or E10 (1/8 dilution with HBSS)). Data are presented as mean ± s.e.m. (n = 3) (**B**) Cortical cells at E13 neocorticies were cultured. At 1DIV RGPs were labeled with BrdU and incubated with artifitial CSF (1/2 dilution with culture media), E10 CSF (1/2 dilution with culture media), or E13 CSF (1/2 dilution with culture media) for 2 days, and immunostained with SOX2 and TUJ1 antibodies. (**C**) The graph showing the fraction of BrdU-positive cells that were also positive for SOX2 or TUJ1 in (**B**). Data are presented as mean ± s.e.m. (n = 4; more than 50 cells were analyzed in each condition). (**D**) Model of GPR157 signaling in RGPs. *p < 0.05, **p < 0.01. Scale bar: 10 μm.

The localization of GPR157 at the cilium exposed to the CSF suggests the possible existence of putative ligand(s) for the orphan receptor in the fluid. To investigate this possibility, we measured the ability of the CSF to activate GPR157 in U-2 OS cells using the Alkaline Phosphatase-TGFα shedding assay^28^. In this GPCR assay system, GPR157 was transfected into U-2 OS cells, together with TGFα whose ectodomain is fused with Alkaline Phosphatase (AP-TGFα). Upon ligand binding to GPR157, endogenous TACE is activated by Gq and cleaves TGFα ectodomain, resulting in AP release into the media. The ratio of AP activity in the media to total AP activity was measured and defined as GPR157-activation index (also called relative AP activity). Using the assay system, we measured the relative AP activity of the CSF collected from the lateral ventricles of embryonic brains at E10 and E13. When E13 CSF was applied to GPR157-transfected and mock-transfected cells, the relative AP activity of GPR157-transfected cells was significantly higher than that of mock-transfected cells (Fig. 5A). On the other hand, when HBSS was applied to the cells, no significant difference in the relative AP activity was found between GPR157- and mock-transfected cells (Fig. 5A). A slightly higher relative AP activity of mock-transfected cells supplemented with E13 CSF was found when compared to that obtained with HBSS (Fig. 5A). This effect is likely due to activation of endogenous GPCR signaling in U-2 OS cells in response to E13 CSF. Importantly, E13 CSF was significantly more potent to activate GPR157 than E10 CSF (Fig. 5A). These results suggest that putative ligand(s) of GPR157 exist in the CSF and that ability of the CSF to activate GPR157 is higher at E13 than at E10. Finally, we examined the neurogenic potential of E13 CSF in RGP cultures. For this purpose, BrdU-labeled RGPs were cultured with either E10 or E13 CSF for 2 days and then immunostained with TUJ1 and SOX2 antibodies. We found that E13 CSF gave rise to more TUJ1-positive neurons (approximately 30%) than E10 CSF (approximately 20%) (Fig. 5B,C). These results indicate that E13 CSF has neurogenic potential more than E10 CSF.

## Discussion

The fate of RGPs is controlled by local environmental signals borne by a variety of sources in a developmental stage-dependent manner. The identification of molecules, their sources and signaling pathways responsible for this control is primordial for our understanding of the mechanisms underlying neurogenesis and brain development.

In the present study, we find that GPR157, an orphan GPCR with no subfamily and splice variant reported to date, is expressed in the developing neocortex. Using a home-made specific antibody (Fig. 1D, Supplementary Figs S1 and S2), we revealed that GPR157 is localized in the primary cilia of RGPs that protrudes into the CSF. Importantly, we found that GPR157 is coupled with Gq-class G-proteins and that GPR157-Gq-IP_3_ signaling contributes to neuronal differentiation of RGPs. This was supported by finding of decreased neuronal differentiation of RGPs *in vivo* upon depletion of GPR157, inactivation of Gq and overexpression of IP_3_ sponge that sequesters IP_3_ and suppress IP_3_-dependent Ca^2+^ release from Ca^2+^ stores. Using cultured primary RGPs we also showed that GPR157/Gq-mediated enhancement of neuronal differentiation was attenuated upon inactivation of Gq and overexpression of IP_3_ sponge. Considering that the main function of IP_3_ is to stimulate Ca^2+^ release from Ca^2+^ stores, our findings suggest that the Gq-PLC-IP_3_-Ca^2+^ pathway elicited by GPR157 contributes neuronal differentiation of RGPs.

Changes in intracellular [Ca^2+^]_i_ in RGPs have been reported to affect their cell-cycle progression and proliferation^18^,^19^,^29^,^30^. For instance, proliferation of RGPs is stimulated by spontaneous Ca^2+^ waves evoked via gap junctions and voltage-dependent Ca^2+^ channels, as revealed by decreased BrdU incorporation upon disruption of Ca^2+^ waves. Also, ATP treatment triggers increased [Ca^2+^]_i_ in RGPs, as this enhances their BrdU incorporation (proliferation) mediated by the metabotropic purine receptor-Gq-IP_3_ pathway^18^. Similarly, PACAP treatment stimulates RGPs proliferation through the Gq-IP_3_ pathway^19^. In our experiments, we found that GPR157-Gq-IP_3_ pathway contributes to neuronal differentiation without affecting BrdU-incorporation in RGPs. Based on these observations, one can suggest RGP’s fate (e.g. proliferation vs differentiation) is determined by distinct GPCR-Gq-Ca^2+^ pathways via different spatio-temporal organization of intracellular Ca^2+^ signals, such as spatial [Ca^2+^]_I_ increase in subcellular compartments or specific temporal patterns of amplitude and frequency of Ca^2+^ transients^31^.

The CSF contains a wide variety of signaling molecules, and its constituents dynamically change throughout brain and spinal cord development^12^. Thus, the CSF is expected to have an active role (e.g., regulation of RGPs biology) beyond providing a fluid cushion for the CNS and maintenance of extracellular ionic balance. In fact, CSF-borne signals have been shown to regulate RGP proliferation^32^. The present study found that GPR157 is present at the primary cilium, and at least one constituents in the CSF can activate GPR157. Intriguingly, its ability to activate GPR157 increases at neurogenic phase (E13), when compared to proliferative phase (E10) (Fig. 5A). This suggests that GPR157-mediated signaling is switched on during the neurogenic phase by virtue of developmentally regulated factor(s) in the CSF yet to be determined. Of note, GPR157 depletion in RGPs at E13 reduced their neuronal differentiation *in vivo* (Fig. 2), but overexpression of GPR157 (GPR157^res^) does not enhance it any further. In contrast, overexpression of GPR157 in cultured RGPs derived from E13 neocortices promoted their neuronal differentiation (Fig. 4E,G). These observations led us to propose the idea that GPR157-mediated signaling is sufficiently activated by CSF factor(s) *in vivo* at E13 but minimal in cultured RGPs devoid of the CSF. In mice, the amniotic fluid is trapped into the neural tube at neural tube closure (around E9) and serves as the initial CSF. Around E11 the choroid plexus starts to develop and produces the CSF^33^,^34^. As the choroid plexus is expected to be a source of many signaling factors^8^,^35^, ligand(s) for GPR157 in the E13 CSF may derive from this secretory structure.

In conclusion, the present study identifies GPR157 located at the primary cilium as a transducer of signals derived from CSF during neurogenic phase, and a modulator for neuronal differentiation of RGPs. Several RGPs-specific GPCRs including GPR157 have recently been identified by RNA-seq^36^,^37^. Further analysis of the GPCR-Gα signaling cascades and the identification of ligand(s) for GPR157 will pave the way for deciphering the complex mechanisms underlying the RGPs-CSF interface in the developing neocortex.

## Methods

### Plasmids

The plasmid expressing GFP under the control of the CAG promoter (pCAGIG) and pCAGEN plasmid that directs cloned gene expression from the CAG promoter were kind gifts from Takahiko Matsuda (Kyoto University, Kyoto, Japan). Plasmid encoding Gαq-CT (expressing Arg306-Val359 of Gαq) in pCAGGS plasmid was a generous gift from Yoh Takuwa (Kanazawa University, Ishikawa, Japan). pBS/U6 plasmid was kindly provided by Yang Shi (Harvard Medical School, MA, USA). pAlph-TGF-alpha plasmid was generously provided by Shigeki Higashiyama (Ehime University, Ehime, Japan).

Plasmid expressing Lifeact-EGFP^21^ was generated by inserting the fragment encoding MGVADLIKKFESISKEE-EGFP into the pCAGEN plasmid. The full-length open reading frame of mouse *Gpr157* was cloned into the pCAGEN plasmid. A plasmid encoding a silent mutant of GPR157 was generated by the QuikChange mutagenesis technique using primers 5′-GCGTCCTTCAGGGCGCTCTATCTACCTTCGCCAACACCAGC-3′ (forward) and 5′-GCTGGTGTTGGCGAAGGTAGATAGAGCGCCCTGAAGGACGC-3′ (reverse). A plasmid encoding Gαq Q209L (CloneID: GNA0Q000C0) was purchased from UMR (University of Missouri-Rolla).

For constructing a plasmid expressing IP_3_ sponge, IP_3_-binding regions of mouse IP_3_R1 (residues 224–604)^27^ was cloned into the pCAGEN plasmid. Plasmids encoding GPR157 shRNA were generated by inserting the annealed oligonucleotides into the pBS/U6 plasmid^38^. Oligonucleotides used were as follows:

GPR157 shRNA #1, 5′-GGTGTCCCGTTGGCCATCACAAGCTTGTGATGGCCAACGGGACACCCTTTTTG-3′ and 5′-AATTCAAAAAGGGTGTCCCGTTGGCCATCACAAGCTTGTGATGGCCAACGGGACACC-3′;

GPR157 shRNA#2, 5′-GGCGCTCTCTCTACTTTCGCAAGCTTGCGAAAGTAGAGAGAGCGCCCTTTTTG-3′ and 5′-AATTCAAAAAGGGCGCTCTCTCTACTTTCGCAAGCTTGCGAAAGTAGAGAGAGCGCC-3′.

### In utero electroporation

DNA solution (~1 μl) in PBS containing 0.02% fast green was injected into the lateral ventricle of the embryos. After injection, electroporation (four 50 ms square pulses of 40 V and 950 ms intervals for E13 embryo, four 50 ms square pulses of 45 V and 950 ms intervals for E14 embryo, four 30 ms square pulses of 45 V and 970 ms intervals for E11 embryo; Nepa Gene, CUY21-EDIT) was carried out with forceps-type electrodes (Nepa Gene, CUY650P5 for E13 and CUY650P3 for E11 and E14) using ICR mice. Final concentrations of the plasmids used were: GFP-expressing plasmid (pCAGIG: 4 μg/μl), Lifeact-EGFP-expressing plasmid (0.8 μg/μl), control shRNA plasmid (pBS/U6: 4 μg/μl), GPR157 shRNA plasmid (4 μg/μl), plasmid expressing silent mutant GPR157 (3.2 μg/μl), GPR157-expressing plasmid (4 μg/μl), Gαq Q209L-expressing plasmid (4 μg/μl) Gαq-CT-expressing plasmid (8 μg/μl and 4 μg/μl for neocortical cell culture) and IP_3_ sponge-expressing plasmid (10.5 μg/μl and 4 μg/μl for neocortical cell culture). For BrdU-labeling experiments, pregnant dams were injected with BrdU (100 mg per gram of body weight at E15) for 30 minutes before harvest. All animal experiments were conducted in accordance with guidelines set by The University of Tokyo and approved (permit number 21-01) by the Committee on Animal Care and Use of the Graduate School of Science in The University of Tokyo.

### GPR157 antibody production and immunostaining

Antibody against GPR157 was generated by Sigma Aldrich as follows. Synthetic peptides corresponding to amino acids Lys314-Ser329 of GPR157 were conjugated to keyhole limpet hemocyanin and used to immunize rabbits. GPR157 antibody was affinity-purified from the serum by a column coupled with synthetic peptides Lys314-Ser329. For immunostaining of E13, E17, P0, and Adult coronal brain sections with anti-GPR157, brains were fixed with 4% paraformaldehyde in PBS overnight at 4 °C. Thereafter, the brain sections (40 μm) were prepared with a vibratome, followed by immunostaining. For immunostaining of E10 coronal brain sections with anti-GPR157, brains were fixed with 4% paraformaldehyde in PBS overnight at 4 °C. Thereafter, the brain sections (40 μm) were prepared with a cryostat, followed by immunostaining. For imaging en face view of the lateral ventricle, telencephalic hemispheres were fixed as describe above and pretreated with HistoVT One solution (Nacalai Tesque) for 15 minutes at 70 °C. Thereafter, the telencephalic hemispheres were immunostained and mounted with the ventricular surface upward.

### Antibodies

Primary antibodies used for immunostaining were as follows: rabbit anti-GPR157 (1:100), mouse anti-ZO1 (1:100, 33-9100; Zymed), mouse anti Ac-tub (1;500, T6793; Sigma Aldrich), rat anti-GFP (1:2000, 04404-84; Nacalai Tesque), rabbit anti-GFP (1:1000, A-11122; Invitrogen), rabbit anti-PAX6 (1:1500, PRB-278 P; Covance), rabbit anti-TBR2 (1:5000, ab23345; Abcam), rat anti-EOMES (1:4000, 14-4875; eBioscience), rabbit anti-SOX2 (1:4000, 23064; Cell Signaling Technology), goat anti-SOX2 (1:200, sc17320; Santa Cruz Biotechnology), mouse anti-TUJ1 (1:5000, MMS-435 P-250; Covance), mouse anti-S100β(1:100, S2532; Sigma Aldrich), mouse anti-DYKDDDDK (1:200, 018-22381; Wako), rat anti-BrdU (1:500, MCA2060T; Bio Rad) and mouse anti-BrdU (1:500, M0744; Dako).

Primary antibodies used for immunoblotting were as follows: rabbit anti-GPR157 (1:1000), rabbit anti-GFP (1:1000, A-11122; Thermo Fisher), and mouse anti-β-actin (1:15000, A1978; Sigma Aldrich).

### Immunohistochemistry

Brain sections were immunostained as described previously^39^. For staining with PAX6, TBR2, SOX2, and S100β brain sections were pretreated with HistoVT One solution for 15–20 minutes at 70 °C. For staining with BrdU, brain sections were pretreated with 4 N HCl for 25 minutes at room temperature. Images were obtained with a 63× objective (Plan-Apochromat, Zeiss) on a Zeiss LSM5 confocal microscope.

### Cell culture, transfection and cell-based analyses

For evaluation of GPR157 knockdown by shRNA constructs, U-2 OS cells maintained in DMEM/10% FBS were transiently transfected using Lipofectamine 2000 (Invitrogen) according to the manufacturer’s instructions. Transfections were allowed to proceed for 4–5 hours, and then cells were cultured in 10% FBS/DMEM for 24 hours. The cells were then subjected to immunoblotting.

For neocortical cell culture, E13 embryos were electroporated with plasmids encoding GFP (pCAGIG), GPR157, Gαq-CT, Gαq Q209L, and IP_3_ sponge. Following electroporation, neocortical cells were prepared as described^40^, plated at a density of ~2.0 × 10^5^ cells on sterile coverslips precoated with Poly-D-lysine in wells of 24-well plates and maintained in DMEM/Ham’s F12 (D-Glucose concentration; 0.6% [w/v]) supplemented with 1% penicillin-streptomycin, 2% B27, 1% N2 and 10 ng/ml bFGF. For analysis of the identity of electroporated RGPs *in vitro*, neocortical cells were fixed at 3 days *in vitro* (DIV3) with 4% paraformaldehyde in PBS for 20 minutes at 37 °C, permeabilized with 0.2% Triton X-100 in PBS for 5 minutes, and blocked with 3% BSA/0.2% Triton X-100 in PBS, followed by incubation with primary antibodies diluted in the blocking solution overnight at 4 °C. The coverslips were then incubated with Alexa 488/Cy3/Cy5-conjugated secondary antibodies overnight at 4 °C and mounted in a Prolong Gold mounting solution. For analysis of CSF’s effect on neuronal differentiation of cultured RGPs, neocortical cells were prepared from E13 brains, plated at a density of ~1.4 × 10^3^ cells in wells 60-well Nunclon Minitray (Nunc) of precoated with Poly-D-lysine. Cells were maintained for 1 day in culture media [DMEM/Ham’s F12 supplemented with D-Glucose (final concentration 0.6% [w/v]), 1% penicillin-streptomycin, 2% B27, 1% N2 and 10 ng/ml bFGF. Thereafter, BrdU (final concentration 10 μM) was added to the media and incubated for 1 hour. The culture media were replaced with artificial CSF (NaCl 119 mM, KCl 2.5 mM, NaHCO_3_ 26 mM, NaH_2_PO_4_ 1 mM, glucose 11 mM, MgCl_2_ 2 mM, CaCl_2_ 2.8 mM) or CSF (1/2 dilution with culture media). Neocortical cells cultured for 2days were then fixed and followed by immunostaining. For BrdU-labeling experiments, BrdU (final concentration 10 μM) was added to the media and incubated for 1 hour before fixation. After fixation, neocortical cells were pretreated with 2 N HCl for 30 minutes at room temperature.

### RT-PCR

Total RNA from mouse neocorticies was prepared by using RNA easy (Quiagen) according to the manufacturer’s protocol. Total RNA was reverse transcribed with Superscript II (Invitrogen) and an anchored (dT)16 primer. The obtained cDNAs were subjected to PCR (Applied Biosystems) by using gene-specific primers.

Primers used were as follows:

Beta-actin-Fw,

5′-CCTTCTACAATGAGCTGCGTGTG-3′;

Beta-actin-Rv

5′-AGAGGCATACAGGGACAGCACAG-3′;

Nestin-Fw

5′-GCTGAGAACTCTCGCTTGCAGAC-3′;

Nestin-Rv

5′-AAGAGAAGGATGTTGGGCTGAGG-3′;

GPR157-Fw

5′-TTGGTCAGAAAGCACATCAACAG-3′;

GPR157-Rv

5′-TGCTTGGTCTCCTAATCCTGAAG-3′.

### Calcium assay

U-2 OS cells were plated at a density of 1.0 × 10^5^ cells in wells of 35 mm glass bottom dish (Matsunami) and maintained in DMEM/10% FBS for 24 hours before transfection. U-2 OS cells were transiently transfected with Lipofectamine 2000. Transfections were allowed to proceed for 4–5 hours, and then cells were cultured in 10% FBS/DMEM. Twenty-four hours later, changes in [Ca^2+^]_i_ were measured by using Screen Quest Rhod-4 NW Calcium Assay Kit (AAT Bioquest) according to the manufacturer’s instructions. In brief, culture medium was replaced with Rhod-4 dye-loading solution, and cells were incubated for 1 hour. Thereafter, the solution was replaced with DMEM without phenol-red after rinsing once. Rhod4 fluorescent images were obtained with 20× objective (EC Plan-Neofluar, Zeiss) on a Zeiss Axio Observer.A1 microscope, and the Rhod4 fluorescent intensity in GFP-labeled cells were measured by using Fiji software. In addition, background fluorescent intensity was also measured in the area with no cells present, was subtracted from Rhod4 measurements. The subtracted Rhod4 measurements were plotted in the graphs.

For analysis of changes in [Ca^2+^]_I_ in cultured RGPs, neocortical cells were prepared from E13 brains, plated at a density of ~2.0 × 10^5^ cells in 35 mm glass bottom dish (Matsunami) precoated with Poly-D-lysine. Cells were maintained for 1 day in culture media [DMEM/Ham’s F12 supplemented with D-Glucose (final concentration 0.6% [w/v]), 1% penicillin-streptomycin, 2% B27, 1% N2 and 10 ng/ml bFGF]. Changes in [Ca^2+^]_i_ were measured as described above.

### AP-TGFα shedding assay

AP-TGFα shedding assay were performed as described previously^27^ with modifications. U-2 OS cells were seeded in wells of 24-well plates at a density of 1.0 × 10^5^ cells/well and maintained in DMEM/10% FBS for 24 hours. The cells were then transiently transfected with plasmids (a mixture of 500 ng/well AP-TGFα plasmid, 150 ng/well GPCR plasmid and 25 ng/well mCherry plasmid) using Lipofectamine 3000. Twenty-four hours later, the cells were rinsed once with PBS, treated with 100 μl of PBS containing 0.05% (w/v) trypsin and 0.20 mM EDTA for several minutes, and suspended with 950 μl DMEM/10% FBS. Small aliquots (105 μl) of the cell suspension were re-seeded in wells of 96-well plates. Twenty-four hours later, the cells were serum-starved for one day with DMEM/0.05%FBS. Thereafter, the cells were rinsed twice with HBSS and then treated with either 30 μl of HBSS or CSF (1:8 dilution with HBSS) for 1 hour at 37 °C. After the incubation, 70 μl of HBSS were added to each well, and 80 μl of the solution were collected into empty wells. p-NPP-containing solution (80 μl) were then added into individual wells. The absorbance at 405 nm was measured immediately and after 1 hour at 37 °C. Relative percentage of AP activity in conditioned medium was calculated as^28^. Samples of CSF were collected from the lateral ventricle as described^41^.

### Statistical analysis

Statistical analyses were carried out with Excel (Microsoft). Sample sizes were based on previous experiments. All data are shown as mean ± s.e.m. Comparison of two groups was analyzed using Welch’s t-test. P < 0.05 was considered statistically significant.

## Additional Information

**How to cite this article**: Takeo, Y. *et al*. The G protein-coupled receptor GPR157 regulates neuronal differentiation of radial glial progenitors through the Gq-IP_3_ pathway. *Sci. Rep*. **6**, 25180; doi: 10.1038/srep25180 (2016).

## Supplementary Material

Supplementary Information
