# Supplementary material for: The G protein-coupled receptor GPR157 regulates neuronal differentiation of radial glial progenitors through the Gq-IP3 pathway
Source: Sci Rep. 2016 May 4;6:25180. doi: 10.1038/srep25180 (PMC4855140; doi:10.1038/srep25180)
Supplement: Supplementary Information [file srep25180-s1.pdf]

The G protein-coupled receptor GPR157 regulates neuronal differentiation of radial glial progenitors through the Gq-IP_3 pathway

Yutaka Takeo[1], Nobuhiro Kurabayashi[2], Minh Dang Nguyen[3] and Kamon Sanada[2*]

SUPPLEMENTARY FIGURES

Supplementary Figure S1

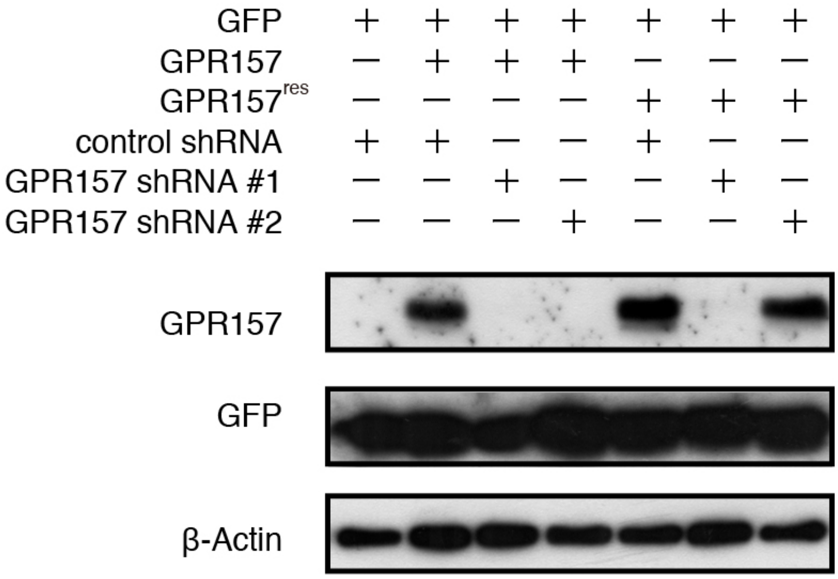

Figure S1. Evaluation of GPR157 shRNA constructs in U-2 OS cells.

U-2 OS cells were transiently transfected with plasmids indicated. The cell lysate was prepared after 24 hours later, and subjected to immunoblotting with antibodies against GFP, GPR157 and β-actin.

Supplementary Figure S2

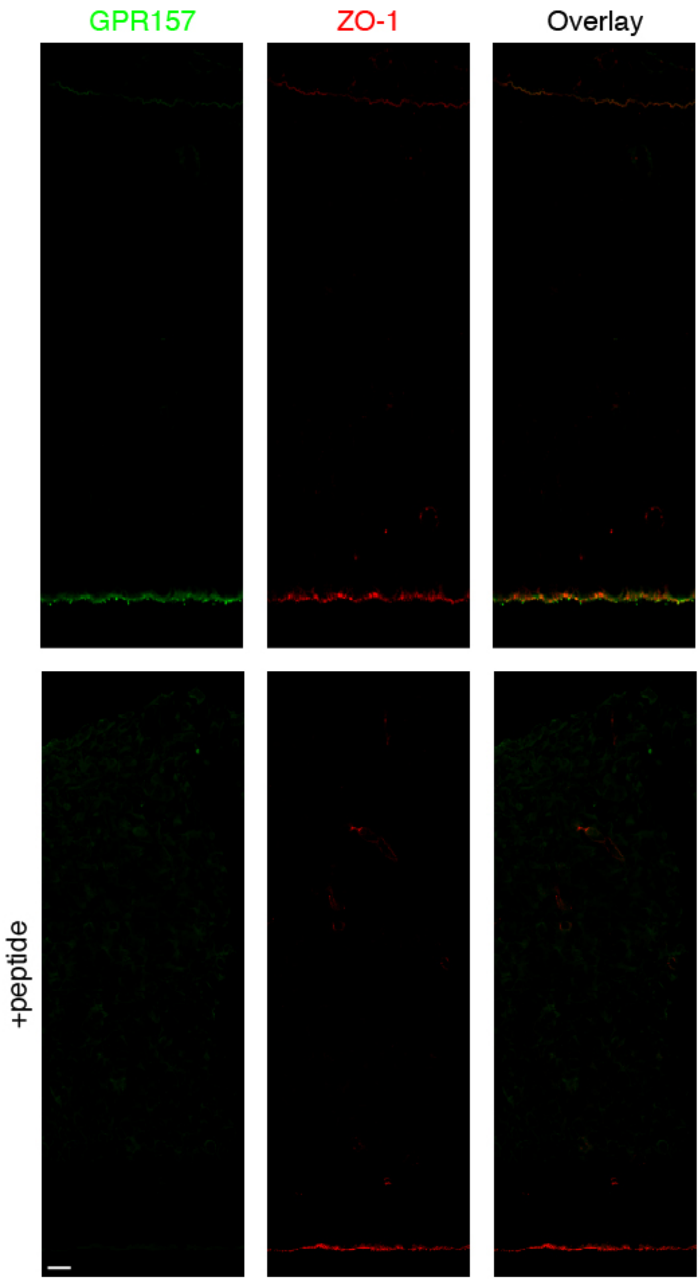

Figure S2. GPR157 immunofluorescent signals are diminished by pre-incubation of the antibody with its immunogen.

Images of E13 neocortical sections immunostained with anti-GPR157 (green) and anti-ZO-1 (red) antibodies. The immunoreactivity of GPR157 is diminished by pre-incubation of the antibody with its immunogen (1.2×10^{-2} mg/ml; lower panels). Scale bar: 10 µm.

Supplementary Figure S3

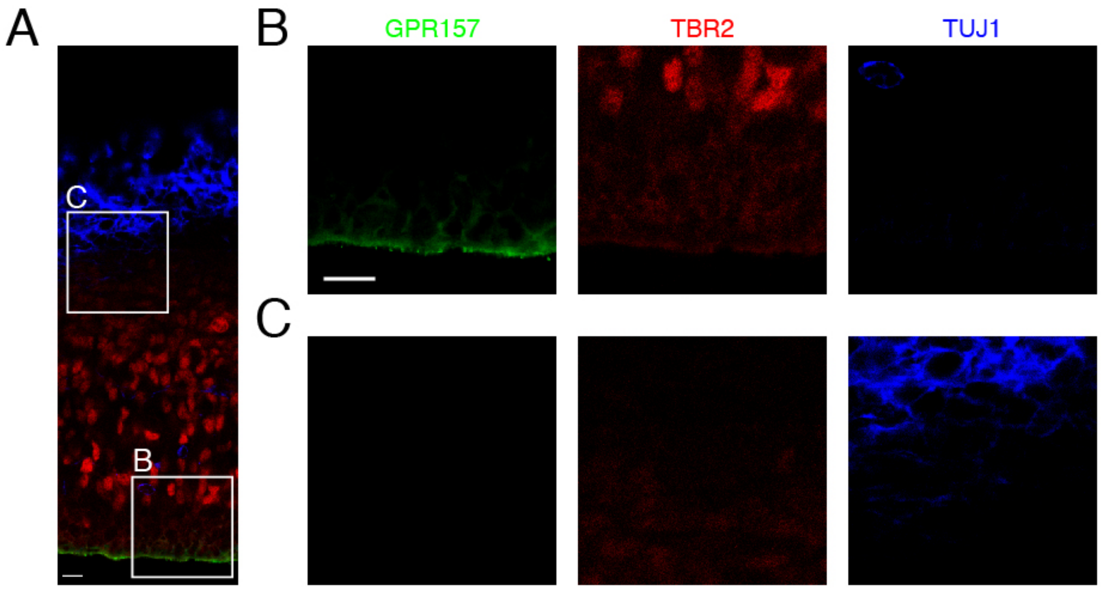

Figure S3. GPR157 immunofluorescent signals were detected at the ventricular surface in the developing neocortex.

(A) An E13 neocortical coronal section immunostained with anti-GPR157 (green), anti-TBR2 (red), and anti-TUJ1 (blue) antibodies. Representative image of the entire cerebral wall is shown. (B, C) Magnified images of the boxed area in (A) are shown. Scale bars: 10 µm.

Supplementary Figure S4

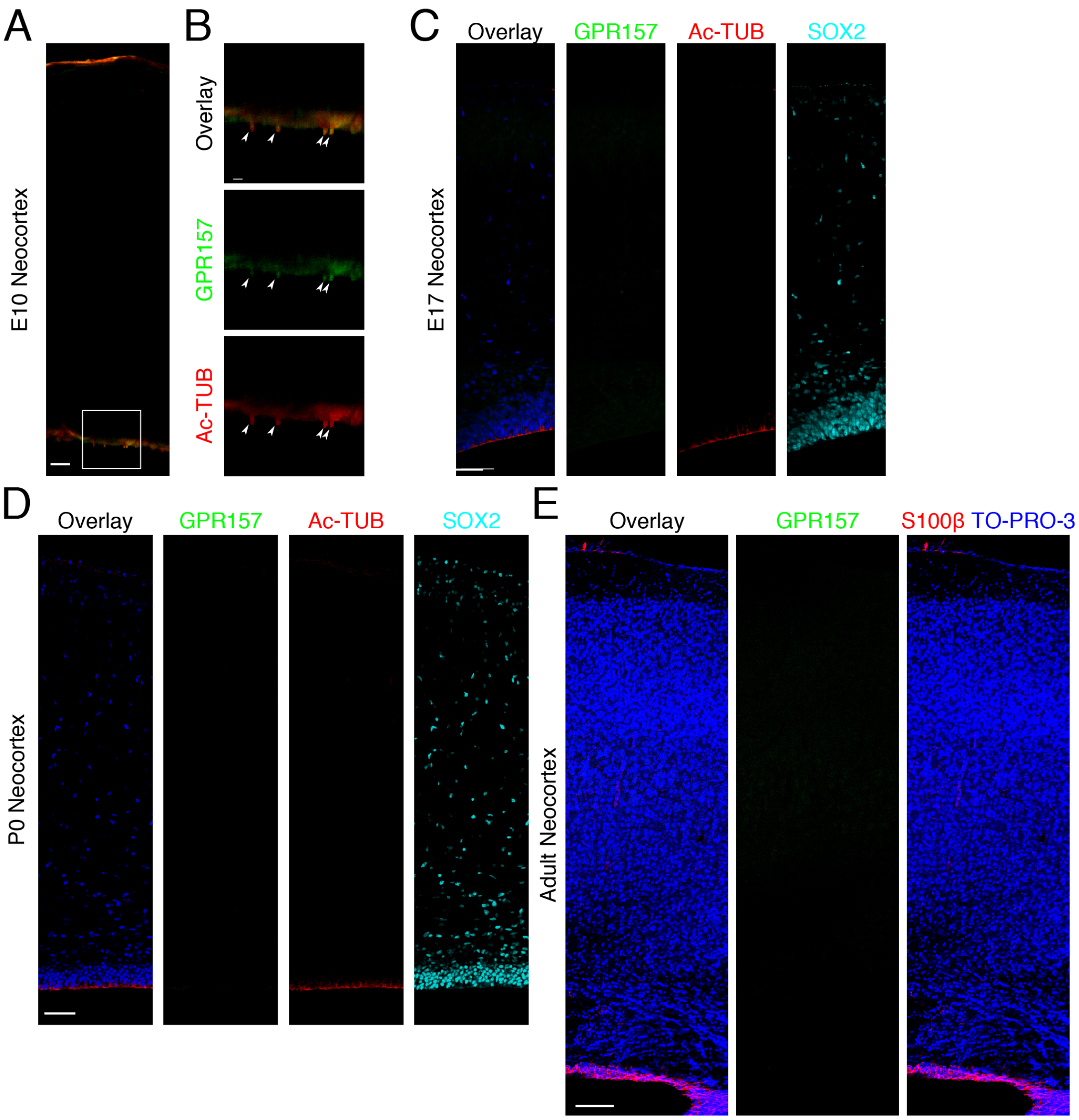

Figure S4. Expression of GPR157 in E10, E17, P0, and adult neocortex.
(A) An E10 neocortical coronal section immunostained with anti-GPR157 (green) and anti-Ac-TUB (red) antibodies. Representative image of the entire cerebral wall is shown. (B) Magnified images of the boxed area in (A) are shown. (C) An E17 neocortical coronal section immunostained with anti-GPR157 (green), anti-Ac-TUB (red), and SOX2 (blue) antibodies. Representative images of the entire cerebral wall are shown. (D) A P0 neocortical coronal section immunostained with anti-GPR157 (green), anti-Ac-TUB (red), and SOX2 (blue) antibodies. Representative images of the entire cerebral wall are shown. (E) An

adult neocortical coronal section immunostained with antibodies against GPR157 (green) and S100β (red), a marker for ependymal cells lining the ventricular surface. Representative images of the entire cerebral wall is shown. Scale bars: 5 µm in A; 1 µm in B; 50µm in C and D; 100µm in E.

Supplementary Figure S5

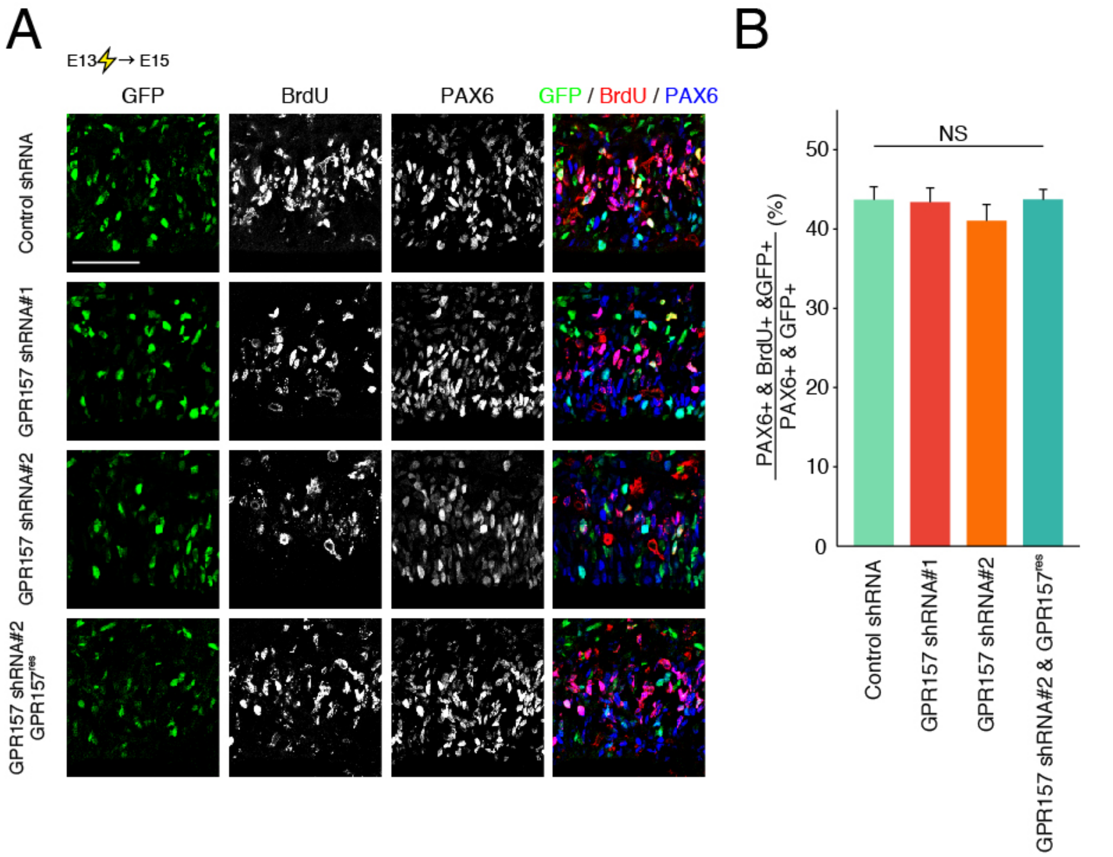

Figure S5. Effect of GPR157 depletion on BrdU-incorporation of RGPs.
(A) Plasmids indicated were electroporated, together with the GFP-expressing plasmid, into E13 embryos as in Figure 2. BrdU was administrated at E15 and the brains were fixed 30 minutes after the BrdU administration. E15 brain sections were immunostained with antibodies against GFP, PAX6, and BrdU. Magnified images around the VZ are shown. (B) The graphs show the fraction of GFP-positive, PAX6-positive RGPs that were also positive for BrdU. Data are presented as mean ±s.e.m (n = 3). NS, not significant. Scale bar: 50 µm.

Supplementary Figure S6

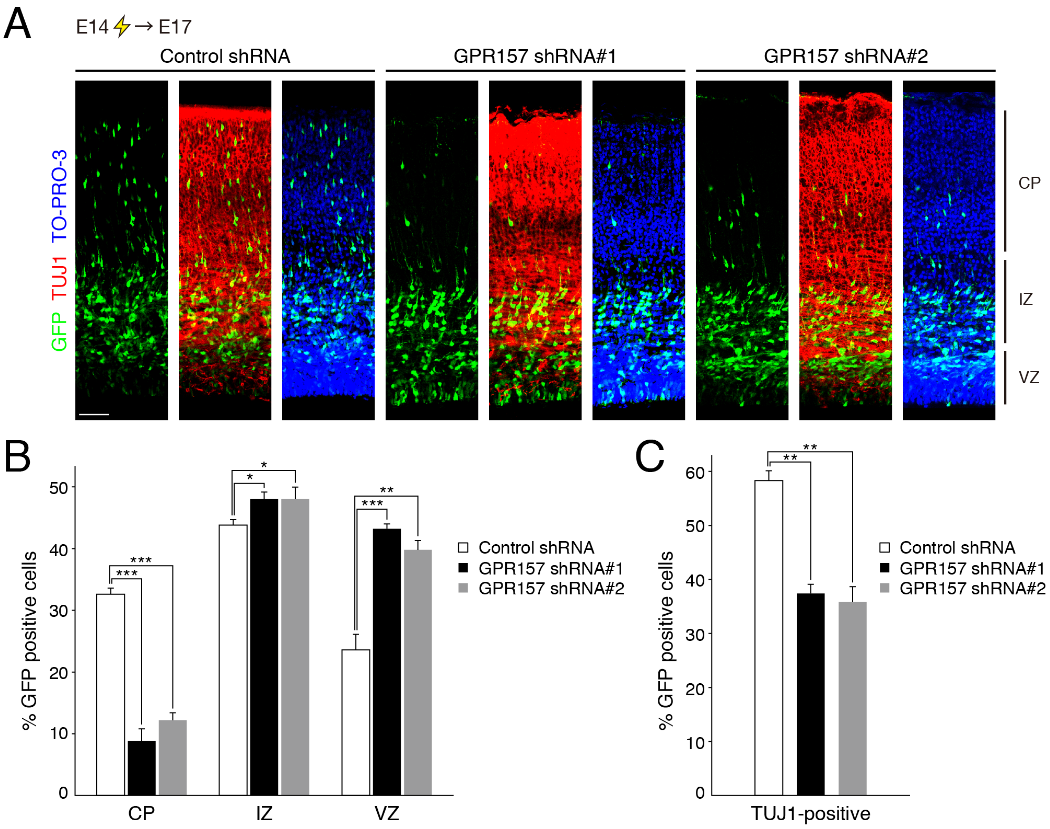

Figure S6. GPR157 depletion in RGPs impairs neuron production.

(A) Plasmids expressing control shRNA, GPR157 shRNA#1, or GPR157 shRNA#2 were electroporated, together with the GFP-expressing plasmid, into E14 embryos. E17 brain sections were immunostained with TUJ1 antibody (red). Images of the entire cerebral wall are shown. Nuclei are stained with TO-PRO-3. (B) Quantification of GFP-positive cell distribution in the CP, IZ and VZ. Data are presented as mean ± s.e.m. (n=3). (C) The graphs show the fraction of GFP-positive cells that were also positive for TUJ1. Data are presented as mean ± s.e.m. (n=3). **p<0.01, ***p<0.001. Scale bar: 50 µm.

Supplementary Figure S7

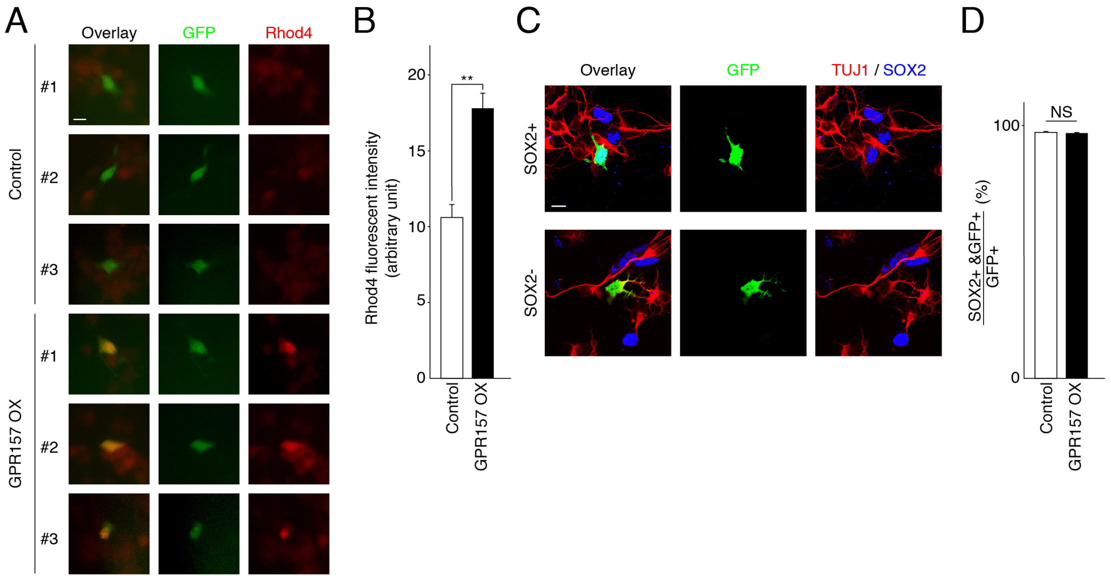

Figure S7. GPR157-mediated signaling increases $[Ca^{2+}]_i$ in RPGs.

(A) Plasmid expressing either GPR157 (GPR157 OX) or control plasmid was electroporated, together with the GFP-expressing plasmid, into E13 embryos. Cortical cells were prepared and cultured for 24 hours. Rhod4, a fluorescent calcium indicator, were used to assess changes in [Ca2+]i. (B) Fluorescent intensity of Rhod4 in GFP-positive cells. Mean ± s.e.m. Data were obtained from 3 independent experiments (more than 100 cells). (C) Examples of GFP-labeled SOX2-positive (upper panels) and SOX2-negative cells (lower panels). (D) The graphs show the fraction of GFP-labeled cells that were also positive for SOX2. Data are presented as mean ±s.e.m. NS, not significant, **$p<0.01$. Scale bars: 10 µm.

Supplementary Figure S8

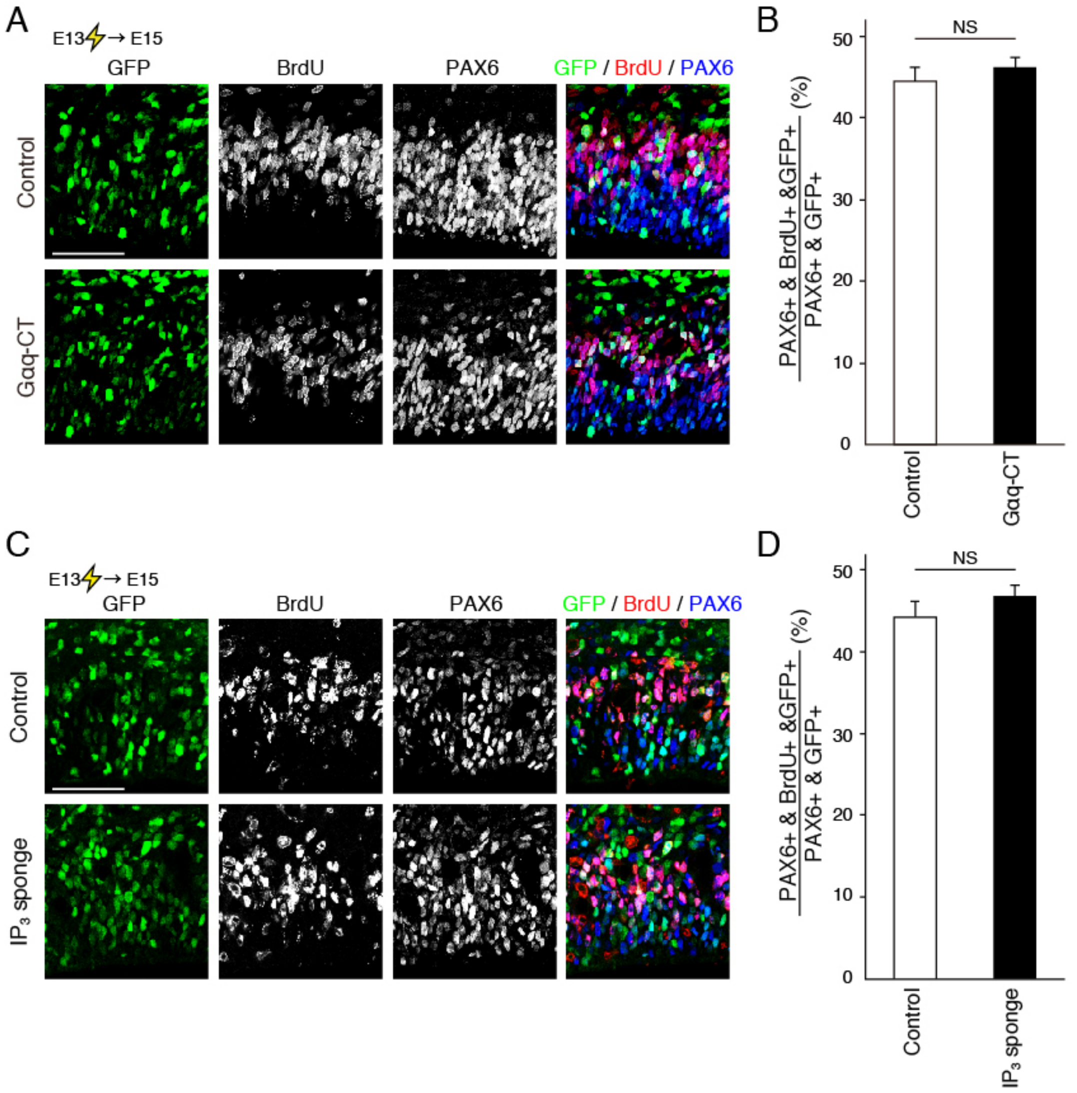

Figure S8. Effect of Gq-CT and IP_3 sponge overexpression on BrdU-incorporation of RGPs.

(A-D) Plasmid expressing either Gq-CT (A, B) or IP_3 sponge (C, D) were electroporated as in Figure 4. BrdU was administrated at E15 and the brains were fixed 30 minutes after the BrdU administration. E15 brain sections were immunostained with antibodies against GFP, PAX6, and BrdU. Magnified images around the VZ are shown. (B, D) The graphs show the fraction of GFP-positive, PAX6-positive RGPs that were also positive for BrdU. Data are presented as mean ±s.e.m (n = 3). NS, not significant. Scale bars: 50 µm.

Supplementary Figure S9

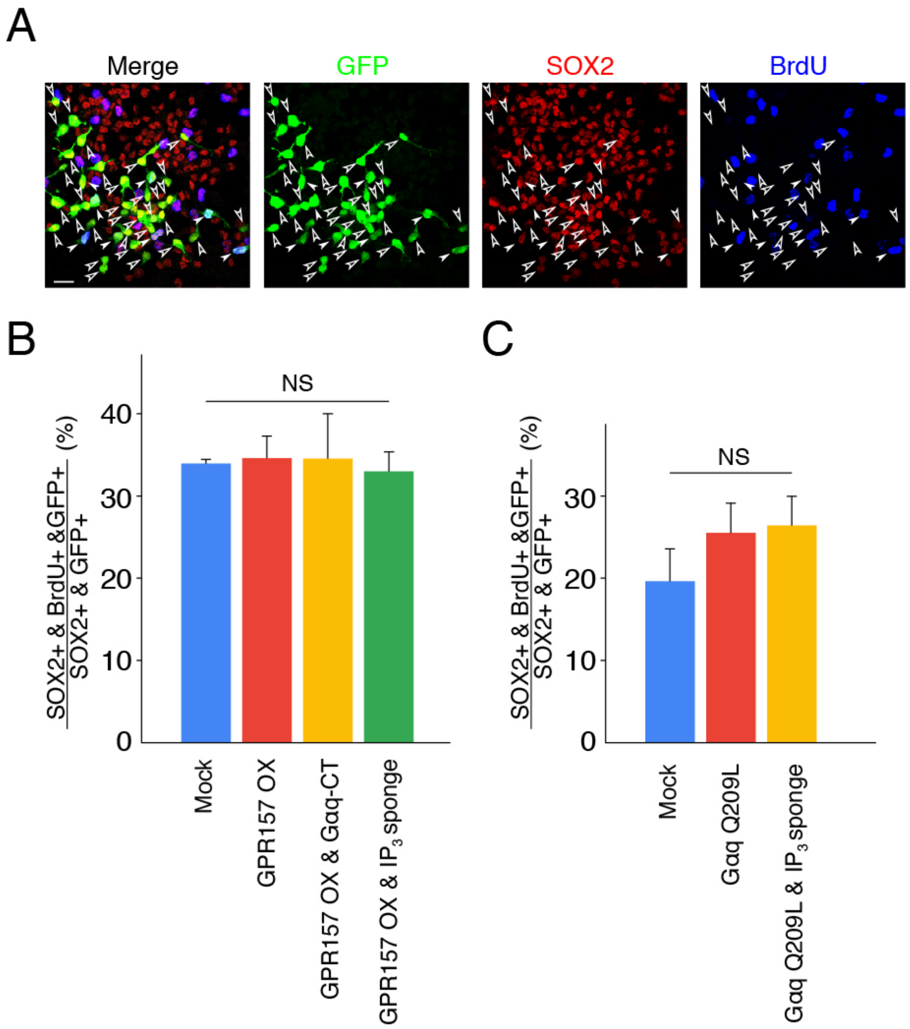

Figure S9. Effect of modulation of the GPR157-Gq-IP_3 pathway in cultured RGPs on BrdU-incorporation.

(A-C) Plasmids expressing a various combination of GPR157, Gαq-CT, IP_3 sponge, and Gαq Q209L were electroporated, together with the GFP-expressing plasmid, into E13 embryos as in Figure 5. Cortical cells were prepared, cultured for 3 days. BrdU was administrated for 1 hour before fixation. The cells were immunostained with antibodies against GFP, SOX2, and BrdU. (A) Examples of BrdU-incorporation in cultured RGPs. Closed and open arrowheads indicate GFP-positive, SOX2-positive RGPs that were also positive for BrdU and negative for BrdU, respectively. (B, C) The graphs show the fraction of GFP-positive, PAX6-positive RGPs that were also positive for BrdU. Data are presented as mean ±s.e.m (n = 3). NS, not significant. Scale bar: 20 µm.
